# Evaluation of Coping Strategies among Students with Type D Personality

**DOI:** 10.3390/ijerph19084918

**Published:** 2022-04-18

**Authors:** Alexey N. Sumin, Ingrid Yu. Prokashko, Anna V. Shcheglova

**Affiliations:** 1 Laboratory of Comorbidity in Cardiovascular Deseases, Federal State Budgetary Scientific Institution “Research Institute for Complex Issues of Cardiovascular Disease”, Sosnovy Blvd., 6, 650002 Kemerovo, Russia; karpav@kemcardio.ru; 2 Federal State-Funded Educational Institution of Higher Education “Kemerovo State Medical University”, Voroshilova Str., 22a, 650029 Kemerovo, Russia; proing59@mail.ru

**Keywords:** personality type D, negative affectivity, social inhibition, coping strategies, stress, medical students, case-control

## Abstract

Objective: Personality type D may be associated with a predisposition to develop stress under external adverse influences, for example, in the COVID-19 pandemic. Likewise, type D personality is associated with higher burnout levels; thus, it may contribute to the development of diseases symptoms. The current study was designed to examine the coping strategies in young healthy persons with personality type D. Methods: The study included 98 medical students, with 30 being males. The participants completed questionnaires to identify personality type D (DS-14) and the coping strategies. Depending on the results of the DS-14 questionnaire, four subgroups were distinguished with different levels of points on the NA and SI subscales. Results: For persons with type D personality, the escape–avoidance strategy was used more often, the accepting responsibility and self-controlling strategies were less common compared with non-type-D individuals. When type D was adjusted for the NA and SI subscales, the correlation remained only with escape–avoidance strategy. We did not find a synergistic effect of the NA and SI subscales in regard to coping. Conclusions: This study demonstrated a link between personality type D and maladaptive coping strategies. The predominance of the maladaptive coping strategy in type D is a possible point of application for psychosocial training in such individuals that requires further research.

## 1. Introduction

The influence of acute and chronic stress on the development and progression of diseases is now well-known [1,2]. Psychological problems can cause distressing reactions; for example, the negative effect of depression on cardiovascular disease is widely known [3,4]. The resistance to the development of depressive and distressed reactions can greatly differ among different persons, thus bringing attention to the concept of personality type D, which reflects a predisposition to the development of psychological distress [5]. Apparently, measures that prevent distressing reactions may be most effective in this cohort. Type D personality is characterized by a combination of two traits: social inhibition (SI) and negative affectivity (NA) [6], which are manifested in various life situations and are fairly stable over time. There is also a genetic predisposition to type D personality [7,8]. Initially, personality type D was actively studied in patients with cardiovascular diseases [6,9,10]. These studies have shown that personality type D has independent unfavorable clinical and prognostic significance [10]. Personality type D is associated with most risk factors for cardiovascular diseases [11], low adherence to treatment and quality of life [9,12], with a number of markers of poor prognosis—for example, the presence of multifocal atherosclerosis [13], calcification of the coronary arteries [14]. Accordingly, personality type D is associated with a poor prognosis in certain categories of patients (with coronary artery disease in younger patients) [15]. There is also a certain geographic diversity in the detection frequency [16] and prognostic value [17,18,19] of type D personality. In further studies, negative associations of personality type D with quality of life and symptom severity in other diseases were confirmed [20,21,22]. Moreover, even in the absence of diseases, personality type D may be associated with a predisposition to develop stress under the external adverse influences, for example, in the COVID-19 pandemic [23,24]. Likewise, type D personality is associated with higher burnout levels [25,26]; it may contribute to the development of stomatognathic disorders’ symptoms [27] and to the development of temporomandibular disorders’ symptoms [28]. However, the most important question, from a practical point of view, is whether it is possible to somehow influence the persons with personality type D and reduce their predisposition to psychological distress and the development of adverse clinical consequences [29,30], and researchers have not yet received an answer to this question. In particular, the point of application of such influences remains unclear. Is it possible to correct the personality type D itself (taking genetic predisposition into account), or is it necessary to increase the subject’s resistance to psychotraumatic situations, looking for options for epigenetic influences? Research (and possible correction) of strategies for overcoming stress in persons with personality type D may be one of the areas of interest. Several recent works have shown that it is the inadequacy of coping strategies that can mediate the adverse effects of personality type D [31], while other studies found no difference in coping strategies [32]. It may be necessary to consider the ethnic, geographic, and cultural characteristics of the patients, thus resulting in research in different regions. This served as the basis for the present study, the purpose of which was to study the choice of coping strategies in individuals with type D and without type D personality in a cohort of young healthy individuals in the Russian sample.

## 2. Methods

### 2.1. Participants

The participants in this study were 98 healthy 2nd-year students (68 females and 30 males) of the Kemerovo Medical University (the level of physical health according to the method Apanasenko [33] was not less than 4 points). The mean age of the participants was 19.8 years (standard deviation, SD = 2.0; range of 18–23). The study was conducted from November 2019 to February 2020, that is, before the start of the COVID-19 pandemic in Russia. All surveys were performed in the laboratory in the morning (from 8:00 to 12:00 a.m.), in the absence of complaints of deterioration in health and decreased performance, and at least 2 h after a light breakfast or fasting, one hour after smoking. Assessments took place individually under the supervision of an investigator (IP) and with the help of senior student volunteers. Filling in self-report questionnaires was the of the data collection. Participants were informed that the questionnaire was voluntary and anonymous; they were assured that there were no correct or incorrect answers and that the answers were confidential. The study was conducted in accordance with the Helsinki Declaration; all students signed an informed consent to the study. The study protocol was approved by the Local Ethics Committee of the FSBSI Research Institute for Complex Issues of Cardiovascular Diseases.

### 2.2. Type D Personality

Evaluation of the psychological status was carried out by using the DS14 questionnaire validated in Russian [34]. The DS14 questionnaire comprises two subscales: negative affectivity (NA) and social inhibition (SI), containing seven questions each. To express agreement/disagreement with each item, a 5-point Likert scale, from 0 (false) to 4 (true), was used. Hence, the total scores for NA and SI subscales range from 0 to 28. If the score was ≥10 points on both subscales, Type D personality was diagnosed. The DS-14 is a valid measure of negative affectivity and social inhibition in the Russian general population [26]. In this study, Cronbach’s alpha for NA was 0.78, and for SI, it was 0.74; that confirms the adequacy of intrinsic structure of Russian version of DS14.

### 2.3. Evaluation of Coping Strategies

To analyze coping strategies, we used the interindividual, multidimensional situation-oriented questionnaire called the Ways of Coping Questionnaire (WCQ), which was developed by Folkman and Lazarus [35] and adapted by Wasserman et al. [36]. The subjects recalled a recent difficult life situation and how they tried to cope with it. The questionnaire contains fifty different options for behavior in a problematic or difficult life situation. Depending on how often the subject uses the described behavior, he or she is offered statements (“never”, “rarely”, “sometimes”, or “often”). These statements are graded on a 4-point system and are grouped into the following scales: confrontational coping, problem-solving planning, self-control, positive reappraisal, taking responsibility, distancing, seeking social support, and escape–avoidance. Confrontational coping involves aggressive behavior in order to change the state of affairs, hostility, and a willingness to take risks. The strategy of planning a solution to a problem is characterized by an activity that includes analysis and development of algorithms for solving a problem. Self-control coping strategies are based on efforts to regulate and control one’s emotions and actions. The positive-reappraisal strategy includes efforts to find positive moments in a problematic or difficult life situation. The coping strategy of taking responsibility is the awareness of one’s own role in the problem and possible ways to solve it. The coping strategy of distancing involves efforts to separate oneself from a problem situation and reduce its significance. Seeking social support is about asking others for help. The coping strategy of escape–avoidance is characterized by efforts aimed at avoiding a difficult life situation. Processing of “raw” indicators was carried out by transferring them to standard T-scores separately for male and female participants, in accordance with age. In addition, the degree of expressiveness of a particular coping strategy for the respondent was determined as rarely used (indicator less than 40 points), moderately used (40 points ≤ indicator ≤ 60 points), or a pronouncedly preferred for the corresponding strategy (indicator over 60 points).

The dominant coping strategies of the individual were determined by the indicator of strategies for overcoming stress (Coping Strategy Indicator, CSI) [37], an adaptation of Sirota et al. [38]. The indicator of strategies for coping with stress allows us to distinguish three groups of fundamental coping strategies: the problem-solving strategy is used when a person identifies a problem and finds effective ways to solve it; the seeking social support strategy is an appeal to others for emotional help (sympathy, understanding), informational help (useful information, advice), and material support; and the avoidance strategy helps reduce emotional stress by avoiding a problem situation. There are 33 judgments in the questionnaire, to which the respondent gives the following answer options: fully agree, agree, and disagree. Answers are scored on a 3-point system. The scales are awarded with different levels of use of the dominant coping strategies of the personality: very low, low, medium, and high.

### 2.4. Statistical Analysis

Statistical processing was performed by using the standard programs Statistica version 10.0 (Dell Software, Inc., Round Rock, TX, USA) and SPSS version 17.0 for Windows (IBM, Armonk, NY, USA). The Kolmogorov–Smirnov test was used to assess the normal distribution of quantitative variables. If the distribution differed from normal, the quantitative indicators were presented in the form of the median and quartiles (25th and 75th percentiles). We applied 2 different analytical approaches to study coping strategies for personality type D. We used the previously established threshold 10 points higher on the social inhibition and negative affectivity scales of the DS14 questionnaire to divide the subjects into four groups: “low negative affectivity/low social inhibition” (NA-SI-), “low negative affectivity/high social inhibition” (NA-SI+), “high negative affectivity/low social inhibition” (NA+SI-), and “high negative affectivity/high social inhibition” (NA+SI+ or Type D). The comparison of quantitative traits between groups was carried out by using the Kruskal–Wallis test. Qualitative and binary characteristics were compared by using the χ^2^ (chi-square) test with Yates’ correction for small samples. Intergroup differences were assessed by using the Mann–Whitney test.

Correlations between indicators of coping-strategy scales (WSQ and CSI scales) and personality-type-D components (NA and SI, type D, and type D adjusted on main effect NA and SI) were analyzed; the relationship between personality-type-D components (NA and SI) and their interaction on indicators of coping-strategy scales (WSQ and CSI scales) was assessed with logistic regression analysis.

To assess the relationship of a binary trait (strong preference of escape strategy in the WSQ scale) with personality-type-D components (NA and SI, NA × SI interaction, and type D), binary logistic regression analysis was used. To identify variables independently associated with strongly preferred escape strategy, the method of stepwise inclusion based on maximum likelihood was used. Additional analysis of the obtained binary classifications was carried out by using ROC curves with an estimate of the AUC indicator. To identify a possible synergistic effect of personality-type-D subscales (NA and SI), we additionally performed linear regression analysis. At the first stage, we identified regression links between the two scales with the indicators of questionnaires for assessing coping strategies. At the second stage, the z-score NA × z-score SI was added to the model of linear regression. The level of critical significance (*p*) was taken as being equal to 0.05.

## 3. Results

### 3.1. Characteristics of the Surveyed Subjects

The general characteristics of the students are presented in Table 1. The surveyed students, according to the results of the DS-14 questionnaire, were divided into four groups, using NA and SI points. The first group consisted of persons who received high scores for NA and low scores for SI (NA+SI-; *n* = 14); the second group was low for NA and high for SI (NA-SI+; *n* = 21); the third group was low in NA and SI (NA-SI-; *n* = 19); and the fourth group was of those who scored above a given level on both grounds, having personality type D (NA+SI+; *n* = 44). The groups were comparable in terms of age and gender.

According to the results of the WSQ questionnaire, the scores on the scale for acceptance of responsibility were significantly higher among students of the first and fourth groups with NA+ than in the groups with NA- (*p* = 0.01). The escape–avoidance scores were also higher for students with type D compared to groups without type D (*p* < 0.001). When analyzing the CSI questionnaire, we noted that only the median of the scores for avoidance strategy in persons with type D was statistically significantly higher than in the other three groups (*p* = 0.04).

### 3.2. Evaluation of Coping Strategies in the Surveyed Groups

Table 2 presents data from the Ways of Coping Questionnaire. The students of the fourth group with type D are characterized by a pronounced preference for the escape–avoidance strategy (75.0%) in comparison with the first group (42.86%), the second group (33.33%), and the third one (21.05%) (*p* = 0.000017). Meanwhile, individuals with non-type D (NA-SI-) used the strategy of acceptance of responsibility reliably less frequently (21.05% of respondents reported “rare use” compared to 2.27% in students with type D (NA+SI+), *p* = 0.022), as well as the self-control strategy (*p* = 0.02 for the trend).

Our evaluation of coping strategies’ peculiarities (Table 3), carried out with the help of the “Indicator of strategies for coping with stress”, notably demonstrated that none of the students with type D reported a high level of problem-solving strategy use (*p* = 0.04 for the trend). The avoidance strategy was reliably more often used among students with type D than among students of the first and third groups (very low level of strategy use in 9.09%, 35.17%, and 47.37%, respectively; *p* = 0.003). The social support seeking strategy showed no statistical difference between the groups.

Association of coping strategies with components of personality type D according to correlation analysis

The correlation analysis (Table 4) revealed a significant relationship between the values of the DS-14 questionnaire on the social suppression scale and scores on the distance, self-control, and escape–avoidance scales of the WSQ questionnaire, and a negative association was only revealed for the strategy for seeking social support of the CSI questionnaire. At the same time, for the values of the negative affectivity scale, an association with points on the distance, self-control, acceptance of responsibility, and escape–avoidance scales of the WSQ questionnaire and the avoidance strategy scale of the CSI questionnaire was noted. The presence of personality type D among students was associated with the following strategies: distance, self-control, acceptance of responsibility, escape–avoidance, and positive reappraisal of the WCQ scale, and the avoidance strategy on the CSI questionnaire scale. However, when type D was adjusted according to the values of the NA and SI scales; the only significant correlation remained with the escape–avoidance strategy.

Association of personality type D components and data from coping-strategies questionnaires according to regression analysis data

We additionally assessed the association of the expressed preference for the escape–avoidance strategy according to the questionnaire WSQ with the components of personality type D using binary logistic regression. When the variables NA, SI, type D, and NA*SI were included in the model (Enter’s method), only type D was the categorical variable that had an association with this indicator (Table 5). In a step-by-step analysis (Forward LR method), subjects with type D were 6.54 times more likely to have pronounced preference for the escape–avoidance strategy than subjects without type D (χ^2^ = 19.08, *p* < 0.001, Nagelkerke R^2^ = 0.236, model correctly classified 71.4% of cases).

**Figure 1:**
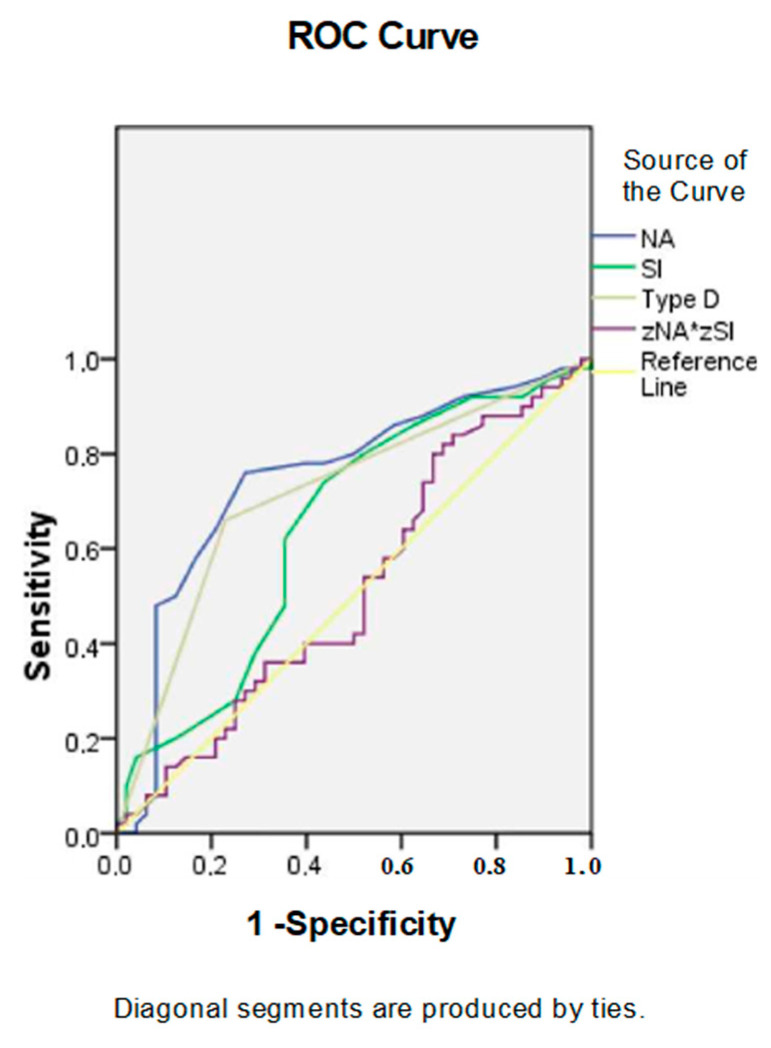
Receiver operating characteristic curve analysis. Performance efficacy of the personality. Type D parameters in the escape–avoidance strategy detecting. Notes: NA, negative affectivity; SI, social inhibition; zNA × zSI, effect of subscales interaction.

In ROC analysis (Figure 1 and Supplementary Table S1), the NA scale values and presence of personality type D (AUC = 0.749 and AUC = 0.715, respectively) predicted to the greatest extent the identification of this strategy by values on the SI scale (AUC = 0.643).

When assessing the correlation of values on the scales of questionnaires and the variable reflecting the interaction of the NA and SI scales, no significant relationships were identified (Table 4). To identify a possible synergistic effect of personality type D subscales (NA and SI), we additionally performed linear regression analysis (Supplementary Table S2). At the first stage, we identified regression links between the two scales with the indicators of questionnaires for assessing coping strategies. Significant associations of the values of the z-score NA scale with points on the acceptance of responsibility (β = 0.335, *p* = 0.001) and escape–avoidance (β = 0.311, *p* = 0.002) scales of the WSQ questionnaire and avoidance strategy (β = 0.212, *p* = 0.043) of the SCI questionnaire were revealed, and associations of the z-score SI scale with points on the self-control (β = 0.244, *p* = 0.019) and escape–avoidance (β = 0.239, *p* = 0.015) scales of the WSQ questionnaire and social support search strategy (β = −0.298, *p* = 0.005) of the CSI questionnaire. At the second stage, the z-score NA × z-score SI was added to the model; no statistically significant effect was obtained for any value of the questionnaire scales. That is, in this case, we were unable to show the synergistic effect of these scales (*p* > 0.05 in all z-score NA × z-score SI cases).

## 4. Discussion

In the present study, the components of personality type D were found to be associated with maladaptive coping strategies. When assessed as a dichotomous variable, a relationship between type D and the following strategies was noted: escape–avoidance (according to the WSQ questionnaire) and avoidance (by the CSI questionnaire). The correction of type D for continuous values of the NA and SI subscales revealed an association with only one coping strategy—the escape–avoidance of the WSQ questionnaire. At the same time, no synergistic influence of the NA and SI subscales on the studied scales of coping strategies was revealed.

Previous studies in persons with personality type D noted numerous variants of maladaptive coping strategies. Studies with United States university students obtained results similar to ours: participants with personality type D reported lower use of positive/problem-focused coping, lower use of social supportive coping, and higher use of avoidant coping than non-Type D subjects [39]. The predominance of avoidance strategies among those with type D helps to understand the results of acute tests with laboratory stressors better. It is not surprising that, with such a prevailing coping strategy, individuals with type D have a less pronounced hemodynamic response under laboratory stress [40,41], but an increase in blood pressure that may persist longer than individuals without type D [42]: avoiding stress can reduce discomfort and physiological arousal in the short term, but this strategy can lead to an inability to cope with stress and difficulty in managing daily life events over time.

Students with personality type D showed significantly higher scores on the burnout-syndrome questionnaire [25], as well as significantly lower levels of stability and a sense of coherence [43]. This is important, since the burnout syndrome has a significant impact on the well-being of students, their academic performance, and the quality of their professional training. The situation has escalated even more against the backdrop of such a highly stressful situation as the COVID-19 pandemic [24,27,28,44]. This is manifested by the high prevalence of moderate and high levels of stress in regard to online learning for students [23], as well as an increased tendency to develop symptoms of diseases. Thus, Gębska et al. [27] showed that, in students with symptoms of disorders of the stomatognathic system, personality type D was detected in 70% of cases, while in the asymptomatic group, only in 23.3%. Among students with personality type D, the most common symptoms of stomatognathic system disorders were headache, pain in the neck and shoulder girdle, and clenching of teeth. Moreover, students with personality type D are more likely to develop temporomandibular disorders and depression during the COVID-19 pandemic [28].

In our study, we did not study burnout syndrome; however, it is known that type D personality correlates not only with burnout syndrome, but also with secondary traumatic stress [45]. It is the tendency to develop psychological distress that is one of the factors in the development and progression of cardiovascular diseases in people with personality type D. For students, the problem of cardiovascular pathology is not relevant, due to their young age; however, the abovementioned problems (burnout syndrome, symptoms of diseases, etc.) can significantly affect academic achievement. It can be emphasized that the prevention of burnout syndrome is especially important both for medical students and subsequently for those working in the medical industry. A significant change in the conditions of study for students (distance learning and volunteer work) and work for medical personnel (work in the “red zone”) in the context of the COVID-19 pandemic creates even more favorable conditions for both psychological distress and the faster development of the burnout syndrome. Persons with personality type D are more prone to developing such reactions; they urgently need the development of preventive programs. As the present study shows, these programs may well be aimed at correcting inadequate coping strategies in students with personality type D.

Previously, the possibilities of psychosocial training in medical university students were studied. This training was aimed at improving the skills that support effective social interaction and communication; it was conducted by a trained psychologist; and it also included group sessions, training in stress management, and coping and relaxation techniques. As a result, the students managed to achieve a significant reduction in the manifestations of the burnout syndrome [46]. It is possible that such skills of coping strategies can later be applied in the workplace, and this can, in practice, prevent burnout syndrome. However, it remains unclear to what extent persons with type D personality will be able to benefit from such psychosocial training; this requires additional research. It also looks promising to use similar approaches for patients with cardiovascular diseases, but such studies have not yet been conducted.

Until now, one of the weak points of the “concept of type D personality” is the lack of understanding of what goals should be directed the psychological or behavioral influences. Indeed, if type D is genetically predetermined and stable in time [8], then how can you influence its manifestations? Taking into account that it was widely believed that type D is characterized by increased stress reactivity [47], the most logical method of influence was to increase stress resistance in persons with type D. The effect of such interventions was controversial. Nyklíček et al. assessed the effect of the 8-week mindfulness-based stress-reduction course in patients with personality type D. In this study, they failed to achieve a decrease in the number of type D patients, despite a slight decrease in the scores on the NA and SI scales [29]. Smith et al. [30] used a special therapeutic technique, “positive emotional writing”, and were unable to reduce the level of anxiety and stress feelings 4 weeks after the intervention in patients with type D. Although the authors were able to show the technique’s effectiveness in the patients with a high level of social suppression and a low level of negative excitability, their belief that this method can be useful for mitigating the negative psychological effects of type D personality in the general population remains questionable [30].

Despite the fact that the first studies of coping strategies in persons with type D were carried out more than 10 years ago, there are still no further studies on the possible modification of these maladaptive strategies.

Moreover, the problem of assessing the “type D personality effect” deserves a separate consideration. The prevailing opinion is that it is wrong to evaluate type D as a dichotomous variable, it is necessary to study the “effect of type D” when assessing continuous values of its subscales, and also to evaluate the synergistic effect of these subscales on the question under study (association with clinical factors, prognosis, etc.) [48,49]. In our opinion, such an approach, while undoubtedly interesting in terms of research, takes us away from providing assistance to our patients. Some of the proposals seem somewhat far-fetched. For example, most studies that tried to identify the so-called “synergistic effect” of type D components failed to do so [19,50,51], not unlike us in this study. At the same time, a fairly extensive collection of the literature on the assessment of the clinical and prognostic value of personality type D when assessing it as a dichotomous indicator convincingly shows its significance. For the practitioner, the facts that persons with type D have a worse quality of life, adherence to treatment, and, ultimately, a poor prognosis are likely far more important than the question of whether the type D subscales have a synergistic effect. In addition, in clinical practice, one often has to make some simplifications and assumptions. For example, in the diagnosis of arterial hypertension, its stage is established at certain levels of systolic and diastolic blood pressure (i.e., instead of continuous variables, a dichotomous one is used). In our opinion, it is quite possible in the future to apply the classical version of the assessment of personality type D, which was shown by both the present study and our previous works in this direction [13,14,18].

### Study Limitations

The study was conducted on a relatively small sample; apparently, confirmation of the data obtained in larger studies is required, with an increase in both the number of participants and the cohorts of the surveyed (older people, with different levels of education, etc.). However, the significant relationship between type D personality and maladaptive coping strategies found in our study confirms the hypothesis of a possible indirect effect of the latter on the diseases development.

The revealed patterns of coping strategies for personality type D in young healthy individuals may differ from those in other cohorts, primarily patients with cardiovascular diseases, in whom the correction of the adverse consequences of having personality type D is most important. Apparently, further studies are required among patients with various pathologies.

Furthermore, the study of personality characteristics in our study was limited only to the assessment of personality type D, since other scales and indicators (for example, Big Five personality traits [52]) were studied quite fully previously.

## 5. Conclusions

Overall, the results of this study demonstrated a link between type D personality and coping strategies in a sample of young healthy Russian individuals. For individuals with type D, the escape–avoidance coping strategy was used more often, and the acceptance of responsibility and the self-control strategies were used less often compared with individuals without type D. The subscales of type D (NA and SI) differed in relation to coping strategies: NA was additionally associated with the strategy of acceptance of responsibility, and SI was associated with the social support search strategy. We have not revealed a synergistic effect of these scales on the studied coping strategies. The predominance of the maladaptive coping strategy escape–avoidance in type D is a possible point of application for behavioral interventions in such individuals, and it requires further research.

## Figures and Tables

**Table 1:** Variables at baseline according to type distress personality DS-14, WSQ/CSI.

Dataset	NA+SI- (*n* = 14)	NA-SI+ (*n* = 21)	NA-SI- (*n* = 19)	NA+SI+ (*n* = 44)	*p*
Mean age, Me (LQ; UQ)	19.0 (19.0; 20.0)	19.0 (19.0; 20.0)	19.0 (19.0)	19.0 (19.0)	0.52
Gender, male (*n*, %)	3 (10.0)	9 (30.0)	9 (30.0)	9 (30.0)	0.08
DS-14					
NA, Me (LQ; UQ), point	13.5 (11.0; 15.0) ^†^	5.0 (4.0; 7.0) ^†,‡^	4.0 (2.0; 6.0) ^†,§^	16.0 (11.0; 18.5) ^‡,§^	<0.001
SI, Me (LQ; UQ),point	8.0 (6.0; 9.0) ^†^	14.0 (11.0; 15.0) ^†,‡^	7.0 (5.0; 8.0) ^‡,§^	13.5 (12.0; 15.5) ^†,§^	<0.001
WSQ					
Confrontational coping, points	59.0 (55.0; 67.0) ^†^	54.0 (47.0; 60.0) ^†^	57.0 (51.0; 63.0)	56.0 (51.0; 67.0)	0.15
Distance, points	55.0 (52.0; 62.0)	59.0 (55.0; 65.0)	55.0 ^§^ (49.0; 59.0)	58.5 ^§^ (55.0; 65.0)	0.1
Self-control, points	55.5 (40.0; 59.0)	55.0 (47.0; 59.0)	50.0 (45.0; 56.0)	55.0 (51.0; 59.0)	0.14
Strong preference for strategy, points	53.5 (48.0; 57.0)	49.0 (40.0; 57.0)	52.0 (43.0; 58.0)	51.5 (47.0; 56.0)	0.7
Acceptance of responsibility, points	60.0 (51.0; 61.0) ^†^	55.0 (52.0; 61.0)	51.0 (43.0; 60.0) ^†,§^	59.5 (55.5; 62.0) ^§^	0.01
Escape–avoidance, points	56.5 (50.0; 63.0) ^†^	56.0 (50.0; 63.0) ^‡^	55.0 (47.0; 59.0) ^§^	66.0 (60.5; 71.0) ^†,‡,§^	<0.001
Problem planning, scores	53.0 (47.0; 63.0)	56.0 (50.0; 59.0)	50.0 (45.0; 59.0)	56.0 (47.0; 59.0)	0.88
Positive revaluation, points	56.0 (53.0; 59.0)	56.0 (53.0; 59.0)	59.0 (51.0; 61.0)	53.0 (48.0; 58.0)	0.25
CSI					
Problem-solving strategy, points	25.0 (22.0; 29.0)	25.0 (22.0; 27.0)	23.0 (21.0; 29.0)	25.0 (20.5; 27.0)	0.67
Social Support Search Strategy, points	22.5 (15.0; 25.0)	19.0 (17.0; 21.0)	22.0 (19.0; 25.0)	22.0 (16.5; 23.0)	0.11
Avoidance strategy, points	18.0 (13.0; 21.0)	18.0 (17.0; 21.0) ^‡^	16.0 (14.0; 18.0) ^§,‡^	20.0 (17.0; 23.0) ^§^	0.04

Note: ^†^ *p* < 0.05 compared to group I; ^‡^ *p* < 0.05 compared to group II; ^§^ *p* < 0.05 compared to group III; NA, negative affectivity sum score; SI, social inhibition sum score; NA-SI-, NA and SI score below cutoff; NA+SI-, NA score above and SI score below cutoff; NA-SI+, NA score below and SI score above cutoff; NA+SI+, NA and SI score above cutoff (type D group).

**Table 2:** Ways of Coping Questionnaire (WCQ), Lazarus.

Dataset	NA+SI- (*n* = 14)	NA-SI+ (*n* = 21)	NA-SI- (*n* = 19)	NA+SI+ (*n* = 44)	*p*
Confrontational coping					
Rare use of strategy	0	1 (4.76)	0	2 (4.55)	0.66
Moderate use of strategy	8 (57.14)	15 (71.43)	12 (63.16)	24 (54.55)	0.6
Strong strategy preference	6 (42.86)	5 (23.81)	7 (36.84)	18 (40.91)	0.55
Distance					
Rare use of strategy	2 (14.29)	0	0	1 (2.27)	0.06
Moderate use of strategy	8(57.14)	12 (57.14)	15 (78.95)	22 (50.0)	0.2
Strong strategy preference	4 (28.57)	9 (42.86)	4 (21.05)	21 (47.73)	0.19
Self-control					
Rare use of strategy	3 (21.43)	0	3 (15.79)	1 (2.27)	0.02
Moderate use of strategy	9 (64.29)	17 (80.95)	15 (78.95)	34 (77.27)	0.6
Strong strategy preference	2 (14.29)	4 (19.05)	1 (5.26)	10 (22.73)	0.39
Seeking social support					
Rare use of strategy	1 (7.14)	5 (23.81)	2 (10.53)	4 (9.09)	0.33
Moderate use of strategy	12 (85.71)	15 (71.43)	14 (73.68)	35 (79.55)	0.74
Strong strategy preference	1 (7.14)	1 (4.76)	3 (15.79)	6 (13.64)	0.6
Acceptance of responsibility					
Rare use of strategy	0	4 (19.05)	4 (21.05) ^§^	1 (2.27) ^§^	0.022
Moderate use of strategy	9 (64.29)	10 (47.62)	11 (57.89)	23 (52.27)	0.77
Strong strategy preference	5 (35.71)	7 (33.33)	4 (21.05)	20 (45.45)	0.3
Escape					
Rare use of strategy	1 (7.14)	0	0	0	0.1
Moderate use of strategy	7 (50.0)	14 (66.67) ^‡^	15 (78.95) ^§^	11 (25.0) ^‡,§^	0.0002
Strong strategy preference	6 (42.86) ^†^	7 (33.33) ^‡^	4 (21.05) ^§^	33 (75.0) ^†,‡,§^	0.00017
Problem planning					
Rare use of strategy	1 (7.14)	2 (9.52)	1 (5.26)	5 (11.36)	0.87
Moderate use of strategy	9 (64.29)	17 (80.95)	15 (78.95)	32 (72.73)	0.68
Strong strategy preference	4 (28.57)	2 (9.52)	3 (15.79)	7 (15.91)	0.52
Positive revaluation					
Rare use of strategy	0	1 (4.76)	0	3 (6.82)	0.51
Moderate use of strategy	11 (78.57)	15 (71.43)	11 (57.89)	33 (75.0)	0.50
Strong strategy preference	3 (21.43)	5 (23.81)	8 (42.11)	8 (18.18)	0.23

Note: ^†^ *p* < 0.05 compared to group I; ^‡^ *p* < 0.05 compared to group II; ^§^ *p* < 0.05 compared to group III; NA-SI-, NA and SI score below cutoff; NA+SI-, NA score above and SI score below cutoff; NA-SI+, NA score below and SI score above cutoff; NA+SI+, NA and SI score above cutoff (type D group).

**Table 3:** Coping Strategy Indicator (CSI), Amirkhan.

Dataset	NA+SI- (*n* = 14)	NA-SI+ (*n* = 21)	NA-SI- (*n* = 19)	NA+SI+ (*n* = 44)	*p*
Problem-solving strategy					
Very low strategy use	0	0	0	2 (4.55)	0.47
Low level of strategy use	2 (14.29)	3 (14.29)	6 (31.58)	10 (22.73)	0.51
Medium strategy use	10 (71.43)	18 (85.71)	12 (63.16)	32 (72.73)	0.44
High level of strategy use	2 (14.29)	0	1 (5.26)	0	0.04
Social support search strategy					
Very low strategy use	0	4 (19.05)	1 (5.26)	4 (9.09)	0.23
Low level of strategy use	4 (28.57)	7 (33.33)	4 (21.05)	12 (27.27)	0.85
Medium strategy use	9 (64.29)	10 (47.62)	11 (57.89)	25 (56.82)	0.79
High level of strategy use	1 (7.14)	0	3 (15.79)	2 (4.55)	0.19
Avoidance strategy					
Very low strategy use	5 (35.17) ^†^	3 (14.29)	9 (47.37) ^§^	4 (9.09) ^†,§^	0.003
Low level of strategy use	7 (50.0)	16 (76.19)	10 (52.63)	32 (72.73)	0.17
Medium strategy use	1 (7.14)	2 (9.52)	0	4 (9.09)	0.59
High level of strategy use	1 (7.14)	0	0	4 (9.09)	0.29

Note: ^†^ *p* < 0.05 compared to group I; ^§^ *p* < 0.05 compared to group III.

**Table 4:** Correlation type D, NA, SI, and WSQ/CSI scales.

Dataset	SI		NA		Type D		Type D **		SI × NA	
	R	*p*	R	*P*	R	*p*	r	*p*	r	*p*
WSQ										
Confrontational coping, points	−0.089	0.386	0.153	0.133	0.063	0.536	0.031	0.763	−0.054	0.601
Distance, points	0.234	0.02	0.237	0.019	0.21	0.038	0.005	0.958	−0.092	0.368
Self-control, points	0.286	0.004	0.213	0.036	0.222	0.028	0.012	0.908	−0.148	0.146
Strong preference for strategy, points	−0.141	0.167	0.106	0.301	0.085	0.407	0.125	0.225	0.021	0.836
Acceptance of responsibility, points	0.183	0.071	0.36	<0.001	0.279	0.005	0.049	0.638	−0.032	0.752
Escape–avoidance, points	0.335	0.001	0.384	<0.001	0.482	<0.001	0.261	0.010	0.082	0.424
Problem planning, scores	−0.03	0.746	0.002	0.987	−0.013	0.902	−0.001	0.991	−0.149	0.142
Positive revaluation, points	−0.189	0.062	−0.099	0.33	−0.214	0.035	−0.134	0.193	−0.138	0.175
CSI										
Problem-solving strategy, points	−0.191	0.06	−0.059	0.563	−0.064	0.53	0.054	0.598	−0.144	0.158
Social Support Search Strategy, points	−0.28	0.006	−0.017	0.865	−0.02	0.845	0.139	0.178	0.099	0.332
Avoidance strategy, points	0.195	0.054	0.252	0.012	0.26	0.01	0.089	0.389	−0.047	0.647

Note: NA, negative affectivity sum score; SI, social inhibition sum score. ** Adjusted for NA and SI.

**Table 5:** Association of the expressed preference for the escape–avoidance strategy according to the questionnaire WSQ with the components of personality type D (binary logistic regression analysis, enter method).

Variables in the Equation							
		B	SE	Wald	df	Sig.	Exp (B)
Step 1 ^a^	NA	0.059	0.044	1.777	1	0.183	1.060
	SI	0.013	0.066	0.038	1	0.845	1.013
	Type D	1.397	0.659	4.496	1	0.034	4.045
	zNA × zSI	−0.041	0.226	0.032	1	0.858	0.960
	Constant	−1.361	0.817	2.773	1	0.096	0.256

Note: NA, negative affectivity; SI, social inhibition; zNA × zSI, interaction of variables z-score NA and z-score SI. ^a^ Variable(s) entered on Step 1: NA, SI, type D, and zNA × zSI.

## Data Availability

Data regarding this manuscript are available in the Federal State Budgetary Scientific Institution “Research Institute for Complex Issues of Cardiovascular Disease”, Kemerovo, Russia.

## Supplementary Materials

The following supporting information can be downloaded at https://www.mdpi.com/article/10.3390/ijerph19084918/s1. Table S1: Receiver operating characteristic curve analysis. Performance of Type D components in discriminating the presence of the pronounced preference for the “Escape-avoidance” strategy; Table S2: Logistic regression analysis of the relationship between personality type D components (HA and SI) and their interaction on indicators of coping strategy scales (WSQ and CSI scales).
