# Supplementary material for: Evaluation of Coping Strategies among Students with Type D Personality
Source: Int J Environ Res Public Health. 2022 Apr 18;19(8):4918. doi: 10.3390/ijerph19084918 (PMC9029841; doi:10.3390/ijerph19084918)
Supplement: Supplementary file 1 [file ijerph-19-04918-s001.zip › ijerph-1568890-supplementary.pdf]

Suppl. Table S1. Receiver operating characteristic curve analysis. Performance of Type D components in discriminating the presence of the pronounced preference for the "Escape-avoidance" strategy.

Area Under the Curve

Test Result Variable(s)	Area
NA	0.749
SI	0.643
Type D	0.715
zNA×zSI	0.518

The test result variable(s): NA, SI, Type D, zNA×zSI has at least one tie between the positive actual state group and the negative actual state group.

Suppl. Table S2. Logistic regression analysis of the relationship between personality type D components (HA and SI) and their interaction on indicators of coping strategy scales (WSQ and CSI scales)

	Model	Unstandardized Coefficients		Standardized Coefficients	T	Sig.	Unstandardized Coefficients		Standardized Coefficients	t	Sig.
		B	Std. Error	Beta			B	Std. Error	Beta		
Confrontational coping	(Constant)	57.337	1.113		51.493	0.000	57.468	1.161		49.491	0.000
	Zscore(NA)	2.212	1.176	0.198	1.882	0.063	2.210	1.181	0.198	1.871	0.064
	Zscore(SI)	0.649	0.515	0.130	1.262	0.210	-1.610	1.188	-0.144	-1.356	0.178
	zNA×zSI						-0.433	1.032	-0.043	-0.420	0.675
Distance	(Constant)	58.316	0.940		62.060	0.000	58.608	0.974		60.145	0.000
	Zscore(NA)	1.755	0.992	0.182	1.769	0.080	1.750	0.991	0.182	1.766	0.081
	Zscore(SI)	1.723	0.992	0.179	1.737	0.086	1.842	0.997	0.191	1.848	0.068
	zNA×zSI						-0.963	0.866	-0.110	-1.112	0.269
Self-control	(Constant)	52.786	0.841		62.754	0.000	53.148	0.867		61.305	0.000
	Zscore(NA)	1.197	0.888	0.138	1.348	0.181	1.190	0.882	0.137	1.350	0.180
	Zscore(SI)	2.119	0.888	0.244	2.386	0.019	2.266	0.887	0.261	2.556	0.012
	zNA×zSI						-1.197	0.770	-0.151	-1.554	0.124
Strong preference for	(Constant)	50.286	0.934		53.815	0.000	50.200	0.975		51.498	0.000
strategy	Zscore(NA)	1.534	0.987	0.164	1.555	0.123	1.536	0.991	0.164	1.550	0.125
	Zscore(SI)	-1.786	0.987	-0.191	-1.811	0.073	-1.821	0.997	-0.194	-1.827	0.071
	zNA×zSI						0.283	0.866	0.033	0.326	0.745
Acceptance of responsibility	(Constant)	54.663	0.994		55.009	0.000	55.129	1.022		53.955	0.000
	Zscore(NA)	3.511	1.049	0.335	3.347	0.001	3.502	1.039	0.334	3.371	0.001
	Zscore(SI)	0.843	1.049	0.081	0.804	0.423	1.033	1.045	0.099	0.988	0.326
	zNA×zSI						-1.538	0.908	-0.161	-1.694	0.094
Escape-avoidance	(Constant)	60.112	0.888		67.692	0.000	59.931	0.924		64.838	0.000
	Zscore(NA)	3.024	0.938	0.311	3.225	0.002	3.028	0.940	0.311	3.221	0.002
	Zscore(SI)	2.327	0.938	0.239	2.482	0.015	2.254	0.945	0.232	2.384	0.019

	zNA×zSI						0.597	0.821	0.067	0.727	0.469
Problem planning	(Constant)	52.745	0.962		54.841	0.000	53.056	0.997		53.226	0.000
	Zscore(NA)	0.122	1.015	0.013	0.120	0.904	0.116	1.014	0.012	0.115	0.909
	Zscore(SI)	-0.350	1.015	-0.037	-0.345	0.731	-0.223	1.019	-0.024	-0.219	0.827
	zNA×zSI						-1.027	0.886	-0.119	-1.160	0.249
Positive revaluation	(Constant)	54.612	0.898		60.791	0.000	54.852	0.933		58.779	0.000
	Zscore(NA)	-0.412	0.948	-0.046	-0.434	0.665	-0.417	0.949	-0.046	-0.439	0.661
	Zscore(SI)	-1.569	0.948	-0.175	-1.654	0.101	-1.471	0.954	-0.164	-1.542	0.127
	zNA×zSI						-0.792	0.829	-0.097	-0.956	0.342
Problem solving strategy	(Constant)	24.500	0.386		63.453	0.000	24.596	0.401		61.283	0.000
	Zscore(NA)	-0.003	0.408	0.000	-0.007	0.994	-0.005	0.408	-0.001	-0.012	0.991
	Zscore(SI)	-0.735	0.408	-0.191	-1.803	0.075	-0.696	0.410	-0.181	-1.696	0.093
	zNA×zSI						-0.316	0.357	-0.090	-0.888	0.377
Social support search strategy	(Constant)	20.724	0.492		42.147	0.000	20.571	0.510		40.345	0.000
	Zscore(NA)	0.371	0.519	0.074	0.715	0.476	0.374	0.518	0.074	0.722	0.472
	Zscore(SI)	-1.500	0.519	-0.298	-2.889	0.005	-1.562	0.521	-0.311	-2.995	0.004
	zNA×zSI						0.506	0.453	0.110	1.117	0.267
Avoidance strategy	(Constant)	18.490	0.487		37.941	0.000	18.545	0.508		36.491	0.000
	Zscore(NA)	1.055	0.515	0.212	2.051	0.043	1.054	0.517	0.212	2.039	0.044
	Zscore(SI)	0.649	0.515	0.130	1.262	0.210	0.672	0.520	0.135	1.292	0.199
	zNA×zSI						-0.184	0.451	-0.040	-0.407	0.685
